# Drug repurposing for Alzheimer’s disease from 2012–2022—a 10-year literature review

**DOI:** 10.3389/fphar.2023.1257700

**Published:** 2023-09-07

**Authors:** Monika E. Grabowska, Annabelle Huang, Zhexing Wen, Bingshan Li, Wei-Qi Wei

**Affiliations:** ^1^ Department of Biomedical Informatics, Vanderbilt University Medical Center, Nashville, TN, United States; ^2^ Departments of Psychiatry and Behavioral Sciences, Cell Biology, and Neurology, Emory University School of Medicine, Atlanta, GA, United States; ^3^ Department of Molecular Physiology and Biophysics, Vanderbilt University Medical Center, Nashville, TN, United States

**Keywords:** Alzheimer’s disease, drug repurposing, validation, real-world data, electronic health records

## Abstract

**Background:** Alzheimer’s disease (AD) is a debilitating neurodegenerative condition with few treatment options available. Drug repurposing studies have sought to identify existing drugs that could be repositioned to treat AD; however, the effectiveness of drug repurposing for AD remains unclear. This review systematically analyzes the progress made in drug repurposing for AD throughout the last decade, summarizing the suggested drug candidates and analyzing changes in the repurposing strategies used over time. We also examine the different types of data that have been leveraged to validate suggested drug repurposing candidates for AD, which to our knowledge has not been previous investigated, although this information may be especially useful in appraising the potential of suggested drug repurposing candidates. We ultimately hope to gain insight into the suggested drugs representing the most promising repurposing candidates for AD.

**Methods:** We queried the PubMed database for AD drug repurposing studies published between 2012 and 2022. 124 articles were reviewed. We used RxNorm to standardize drug names across the reviewed studies, map drugs to their constituent ingredients, and identify prescribable drugs. We used the Anatomical Therapeutic Chemical (ATC) Classification System to group drugs.

**Results:** 573 unique drugs were proposed for repurposing in AD over the last 10 years. These suggested repurposing candidates included drugs acting on the nervous system (17%), antineoplastic and immunomodulating agents (16%), and drugs acting on the cardiovascular system (12%). Clozapine, a second-generation antipsychotic medication, was the most frequently suggested repurposing candidate (N = 6). 61% (76/124) of the reviewed studies performed a validation, yet only 4% (5/124) used real-world data for validation.

**Conclusion:** A large number of potential drug repurposing candidates for AD has accumulated over the last decade. However, among these drugs, no single drug has emerged as the top candidate, making it difficult to establish research priorities. Validation of drug repurposing hypotheses is inconsistently performed, and real-world data has been critically underutilized for validation. Given the urgent need for new AD therapies, the utility of real-world data in accelerating identification of high-priority candidates for AD repurposing warrants further investigation.

## 1 Introduction

Alzheimer’s disease (AD) is the most common cause of dementia among older adults, affecting an estimated 6.7 million individuals age 65 and older in the United States (US) in 2023, with a projected prevalence of 13.8 million by 2060 (Alzheimer’s and Dementia, 2023). Currently, only seven drugs are approved by the U.S. Food and Drug Administration (FDA) for the treatment of AD: three cholinesterase inhibitors (donepezil, rivastigmine, galantamine); a glutamate regulator (memantine); a combination of a cholinesterase inhibitor and glutamate regulator (donepezil/memantine); and two amyloid β-directed monoclonal antibodies (aducanumab and lecanemab) (Medications for Memory Loss, 2022). To date, limited progress has been made in developing new treatments for AD. From 2003 to 2022, only one new drug, aducanumab, was controversially approved for use in AD (Mukhopadhyay and Banerjee, 2021). The most recently approved drug, lecanemab, was granted accelerated FDA approval for AD treatment in January 2023 despite controversy—although lecanemab showed modest success in slowing cognitive decline, it also raised safety concerns related to serious adverse events such as brain swelling and hemorrhage (van Dyck et al., 2022). Lecanemab has since received full FDA approval.

New AD drug development has faced many obstacles, including high monetary and time costs and high failure rates in preclinical and clinical trials (Cummings et al., 2014; Yiannopoulou et al., 2019). The complex and incompletely understood pathogenesis of AD, along with its heterogeneous clinical presentation and numerous associated comorbidities, have made developing new therapies for AD an incredibly challenging endeavor. In light of these difficulties, researchers have begun to pursue additional strategies to identify potential treatments for AD. Drug repurposing, the process of exploring additional uses for existing drugs, represents one of such supplemental strategies. Drug repurposing offers several advantages compared to new drug development, including reduced development time frames, lower costs, and importantly, increased assurance of drug safety (Pushpakom et al., 2019), making it an attractive approach for investigating potential AD treatments.

Throughout the last decade, countless drugs have been suggested for repurposing in AD using a wide range of approaches. This review aims to understand the current status of drug repurposing for AD, including the progress made in identifying drug candidates and how the research has changed over time. We mined the scientific literature for AD drug repurposing studies published between 2012 and 2022, and appraised the drug repurposing strategies employed and the drug candidates proposed. Importantly, we examined how studies validated the efficacy of proposed candidates, particularly in terms of validation quality and relevance of validation data to AD, which has not previously received attention. We hope that this review can provide insight into promising repurposing candidates for AD, as well as the data used to suggest and support their therapeutic potential.

## 2 Materials and methods

To identify articles for review, we queried the PubMed database using a full-text search (i.e., search not restricted to title/abstract) with the keywords “Alzheimer’s” AND “drug repurposing”. We limited the search to articles published within the last 10 years (May 2012-May 2022). This query returned 353 results (Supplementary Table S1). Lifting the search time restriction yielded only seven additional results; therefore, our analysis focused on the articles published in the last decade.

### 2.1 Eligibility criteria

**FIGURE 1:**
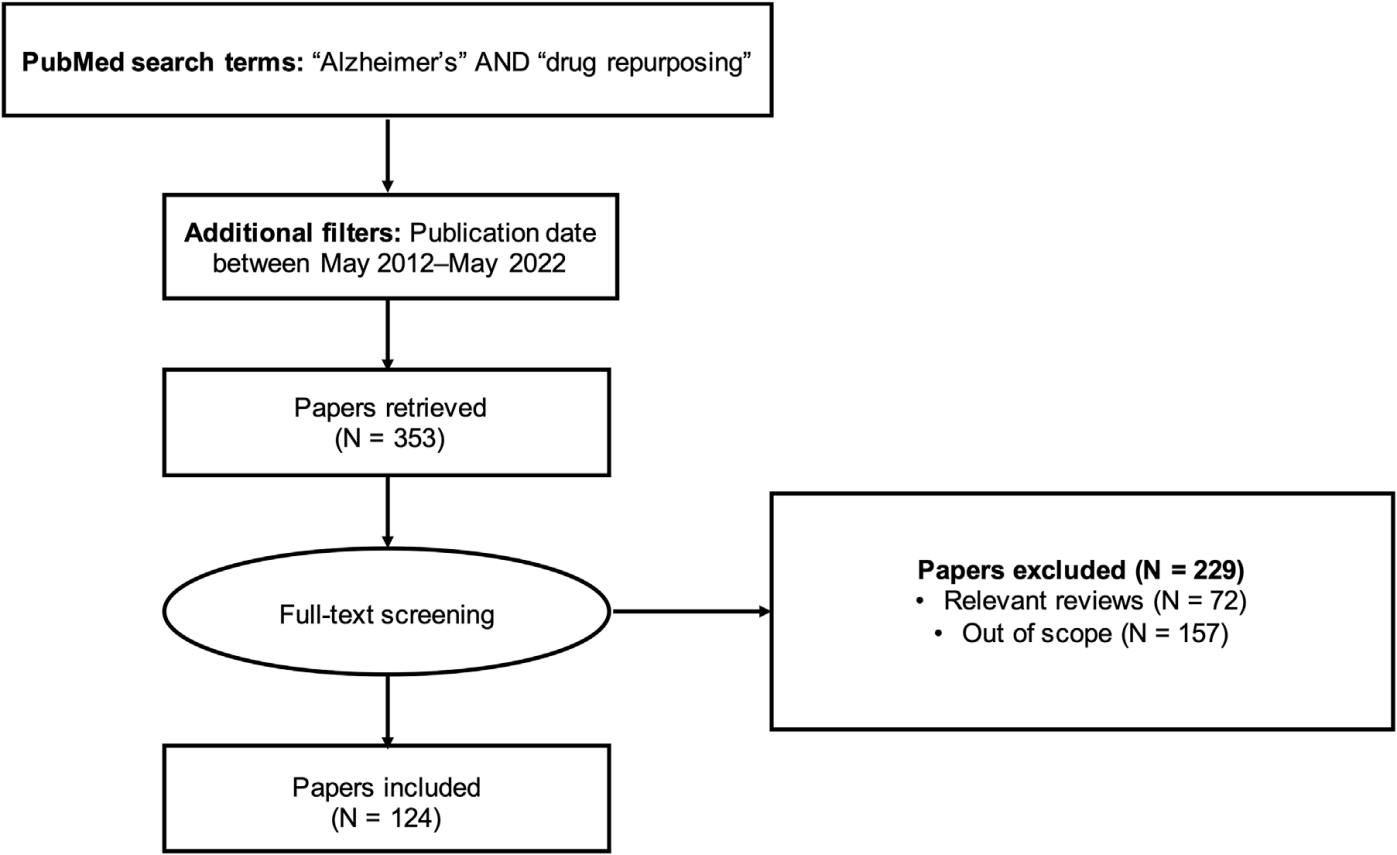
Flow diagram for literature review.

We manually screened the 353 articles and excluded review articles (N = 72) and out-of-scope articles (N = 157). The literature review workflow is summarized in Figure 1. Articles outside the scope of the research question included studies investigating drug repurposing opportunities for diseases other than AD, studies focusing on designing new compounds or modifying existing compounds (for example, synthesizing and evaluating nitazoxanide-based derivatives for potential use in AD treatment (Li et al., 2020) or preparing and assessing a novel ibuprofen microemulsion (Wen et al., 2021)), studies suggesting possible gene targets for AD therapies without mention of existing drugs acting on those targets, and study protocols and other descriptions of planned work. We applied a broad definition of AD in identifying relevant studies, including not only those studies with the primary aim of identifying drug repurposing candidates for AD, but also studies with indirect relationships to AD. For instance, we included studies developing general drug repurposing tools and making predictions for AD (Wu et al., 2013; Croset et al., 2014; Issa et al., 2016; Liu et al., 2016; Jiang et al., 2019; Yu and Gao, 2019; Zeng et al., 2019; Cai et al., 2021; Chen et al., 2021; Tsuji et al., 2021; Muslu et al., 2022), as well as studies suggesting drug repurposing candidates with uses not limited to AD (for example, drugs with potential for treating both AD and depression (Fukuchi, 2020)).

### 2.2 Data extraction

After removing non-qualified articles, we included 124 relevant articles for this review. For each of the 124 studies, we collected information on the repurposing strategy, suggested drug repurposing candidates, and validation plan. The type of repurposing strategy (experimental, computational, or combination) was identified based on the preliminary evidence used to suggest drug candidates (e.g., *in vitro* screening and *in vivo* assays were considered experimental drug repurposing approaches; virtual screening, network models, machine learning models, gene signatures, and data mining were considered computational drug repurposing approaches). The seven drugs currently approved for the treatment of AD (donepezil, rivastigmine, memantine, galantamine, memantine/donepezil, aducanumab, and lecanemab) were excluded from the analysis.

For each study, we documented positive and negative findings. We considered positive findings to be drugs reported as potentially effective in AD (with or without validation of these effects). We interpreted negative findings as drugs found to increase AD risk or drugs with no impact on AD risk, depending on the study authors’ interpretation provided in the discussion of the study findings. We did not independently attribute negative findings based on the study validation (or lack thereof).

We also recorded the journal that each study was published in and the publication year. We collected the most recent impact factor of each journal from Journal Citation Reports (JCR); for journals without an impact factor in JCR, we used the most recent Scopus 2-year impact score, as JCR and Scopus impact factors have been found to be similar (Gray and Hodkinson, 2008). One journal did not have an impact factor available (*Nature Aging*).

The data extracted during literature review is available in Supplementary Table S2.

### 2.3 Characterization of validation

We defined validation as confirmation of a previously identified signal using an intentionally designed study with well-defined outcome measures. We categorized the validation as *in vitro*, *in vivo*, or other. *In vivo* validation included animal models, randomized controlled trials, and real-world data (defined as patient data collected outside of clinical trials—e.g., data from electronic health records [EHRs] and health insurance claims data (Park, 2021)). The “other” category of validation included computational experiments such as molecular docking simulations or queries of drug perturbation databases. We did not consider use of existing literature support to fall within our definition of validation, as this shows replication of results rather than true validation. We also did not consider gene set enrichment analysis and pathway analysis to serve as validation, requiring a more direct link between the suggested drugs and AD to be considered validation. For studies with validation, we assessed whether the validation was specific to AD, defined as validation studies using cellular or animal models of AD or evaluating clinical outcomes of AD. Validation not specific to AD included animal or cellular models unrelated to AD and molecular investigations of proposed drugs.

### 2.4 Drug standardization and classification

We used RxNorm (Nelson et al., 2011), a standardized nomenclature for clinical drugs maintained by the National Library of Medicine, to normalize the names of the drug repurposing candidates so that all drugs were referred to by their generic names. As drug repurposing involves identifying novel therapeutic applications for existing drugs, we used the Prescribable RxNorm API (Prescribable RxNorm API, 2022) to identify the currently prescribable drugs among the suggested drug candidates. We first mapped drug names to RxNorm Concept Unique Identifiers (RxCUIs). To account for differences in reporting of drug names across studies (for example, two studies suggesting different salt forms of the same drug), we mapped the drug RxCUIs contained in the RxNorm Prescribable subset to their ingredient RxCUIs. For example, we mapped the drug valproic acid (RxCUI = 11118) to its ingredient valproate (RxCUI = 40254). Similarly, we mapped the drug glatiramer acetate (RxCUI = 84375) to its ingredient glatiramer (RxCUI = 214582). While some drugs map to multiple ingredients in RxNorm, we only encountered drugs that had a one-to-one mapping between the original drug RxCUI and the ingredient RxCUI.

We then used the Anatomical Therapeutic Chemical (ATC) Classification System to group the drugs at five different levels (Anatomical Therapeutic Chemical ATC, 2022). The Level 1 ATC codes group drugs into fourteen groups based on anatomical system of action (e.g., C for cardiovascular system) and pharmacological properties (e.g., L for antineoplastic and immunomodulating agents); ATC Levels 2–5 further categorize drugs into therapeutic, pharmacological, and chemical subgroups. We extracted ATC codes for the ingredient RxCUIs using the RxNorm API. The drug-ATC code mapping is a one-to-many relationship, as a single drug with multiple therapeutic uses can map to multiple ATC codes.

## 3 Results

**FIGURE 2:**
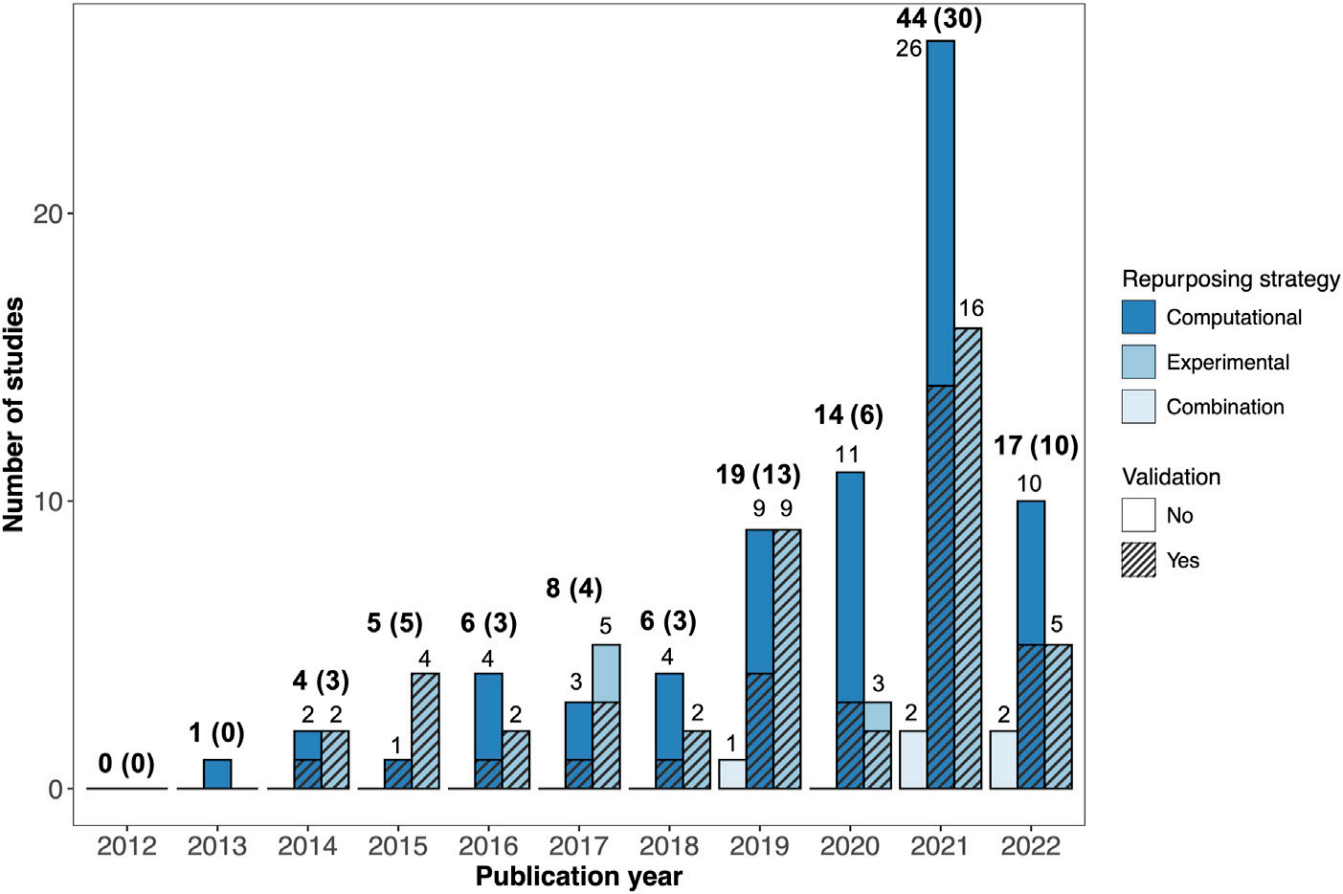
Breakdown of AD drug repurposing studies published between 2012 and 2022. The studies are colored by repurposing strategy (computational, experimental, or combination). Studies with validation are shown with hatching. The total number of studies published each year is shown in bold text, with the number of studies with validation provided in parentheses.

The number of AD drug repurposing publications increased dramatically over the last decade. No primary drug repurposing studies were published in 2012 and only one study was published in 2013, compared with 44 studies published in 2021 (Figure 2).

### 3.1 Drug repurposing approaches

The drug repurposing strategies utilized by the reviewed studies varied widely. Out of the 124 studies reviewed, 71 employed computational (*in silico*) repurposing strategies, 48 involved experimental repurposing approaches (*in vitro* and/or *in vivo*), and five used a combined approach (Figure 2). There was a marked increase in the number of studies using computational drug repurposing strategies between 2012 and 2022, with 26 computational studies published in 2021, compared to sixteen experimental studies and two combination studies.

Out of the 48 studies using experimental drug repurposing approaches, 22 used *in vitro* methods and 20 used *in vivo* methods, while the remaining six studies used a combination of the two. Out of the computational drug repurposing studies, network modeling was the most common approach (N = 21 studies). Other common computational approaches were machine learning (N = 15), genetic signatures (N = 14), structure-based analyses (N = 14), and non-NLP data mining (N = 13). Mendelian randomization was the most infrequently used computational approach (N = 4). Eight studies used a combination of different computational approaches.

We found that most of the reviewed studies (98/124) focused exclusively on drug repurposing for AD, with the exception of eleven studies which developed general tools for drug repurposing and fifteen studies which suggested drug repurposing candidates for other diseases in addition to AD. The studies that used computational repurposing approaches identified an average of 13 ± 18 drug candidates, compared to an average of 2 ± 2 drug candidates for experimental studies and 9 ± 9 drug candidates for combination studies.

#### 3.1.1 Frequently suggested drugs

**FIGURE 3:**
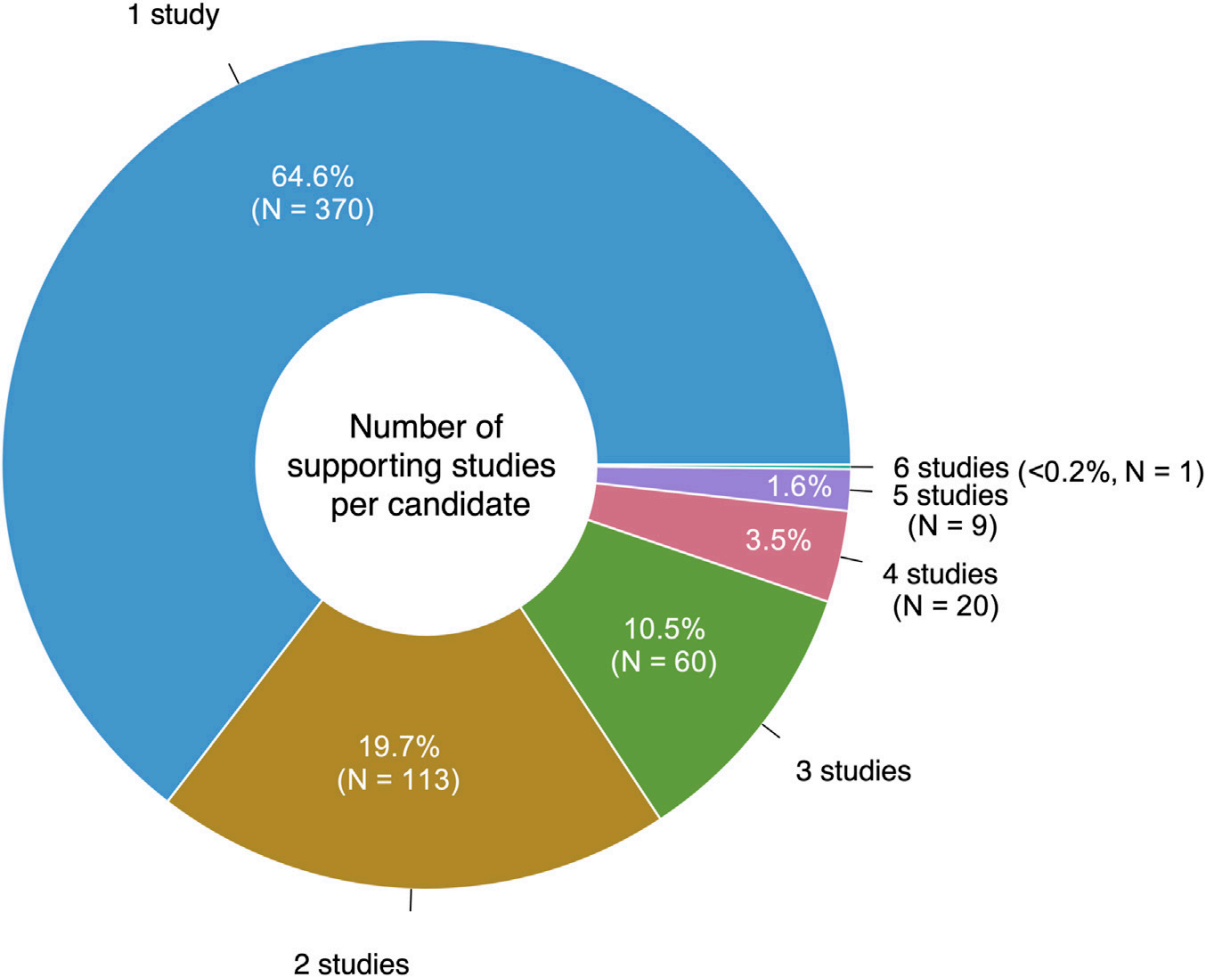
Number of unique studies supporting each drug repurposing candidate.

Eighteen of the reviewed studies proposed only preclinical drugs and other non-prescribable drugs, which were excluded from our analysis. Still, 106/124 (85%) of the reviewed studies proposed at least one prescribable drug candidate, with a total of 573 unique drugs suggested over the 10-year period. 65% (370/573) of these drugs were only suggested by a single study (Figure 3). Notably, eight studies suggested drug combinations rather than single drugs.

The most frequently suggested AD repurposing candidate was clozapine, a second-generation antipsychotic, which was proposed by six unique literature studies. Nine drugs had five supporting studies: the second-generation antipsychotic medications aripiprazole and risperidone, the tyrosine kinase inhibitors sunitinib and vandetanib, the histone deacetylase inhibitor vorinostat (used to treat cutaneous T-cell lymphoma), the antidiabetic drug pioglitazone, the selective estrogen receptor modulator tamoxifen, the calcium channel blocker and antihypertensive medication verapamil, and adenosine (used in treating certain types of cardiac arrhythmia). These top ten highly suggested drugs are described further in Table 1.

**TABLE 1:** Frequently suggested AD repurposing candidates. The number of supporting studies and Level 1 and Level 3 ATC classifications are indicated for each drug.

Drug	Number of supporting studies	ATC classification (Level 1)	ATC classification (Level 3)
Clozapine	6	Nervous system	Antipsychotics
Adenosine	5	Cardiovascular system	Other cardiac preparations
Aripiprazole	5	Nervous system	Antipsychotics
Pioglitazone	5	Alimentary tract and metabolism	Blood glucose lowering drugs, excluding insulins
Risperidone	5	Nervous system	Antipsychotics
Sunitinib	5	Antineoplastic and immunomodulating agents	Protein kinase inhibitors
Tamoxifen	5	Antineoplastic and immunomodulating agents	Hormone antagonists and related agents
Vandetanib	5	Antineoplastic and immunomodulating agents	Protein kinase inhibitors
Verapamil	5	Cardiovascular system	Selective calcium channel blockers with direct cardiac effects
Vorinostat	5	Antineoplastic and immunomodulating agents	Other antineoplastic agents

#### 3.1.2 Drug classification

**FIGURE 4:**
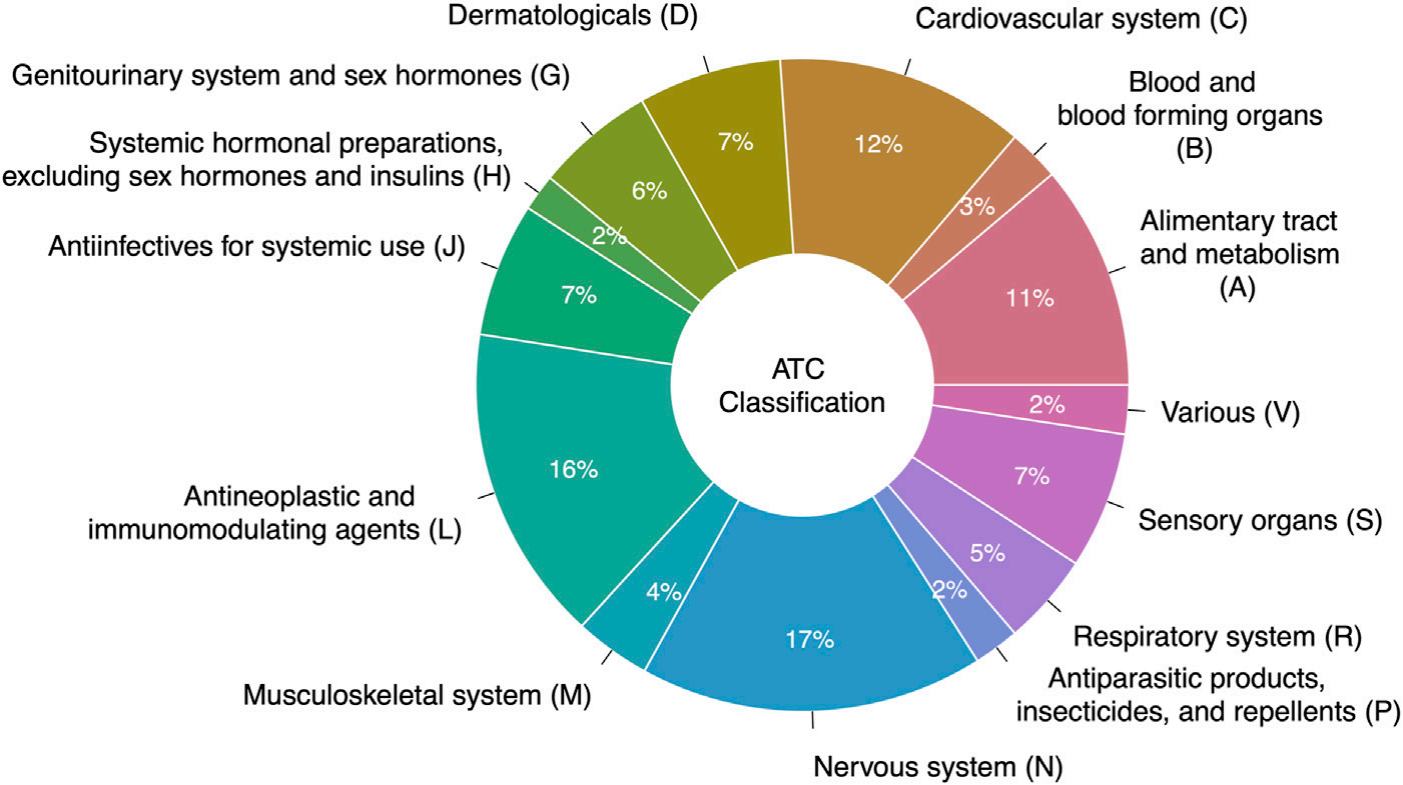
Original therapeutic indications of drugs suggested for repurposing in AD. Drug therapeutic indications are represented using their Level 1 ATC codes.

We mapped 531 of the 573 prescribable drugs to their ATC codes (42 drugs did not have an ATC code). A single drug can map to multiple ATC codes; we took all possible mappings into consideration. As shown in Figure 4, the proposed AD repurposing candidates fell within a variety of anatomical and pharmacological classifications (based on Level 1 ATC code), with drugs acting on the nervous system (ATC code N), antineoplastic and immunomodulating drugs (ATC code L), and drugs acting on the cardiovascular system (ATC code C) cumulatively comprising nearly half of the suggested candidates (17%, 16%, and 12%, respectively).

**FIGURE 5:**
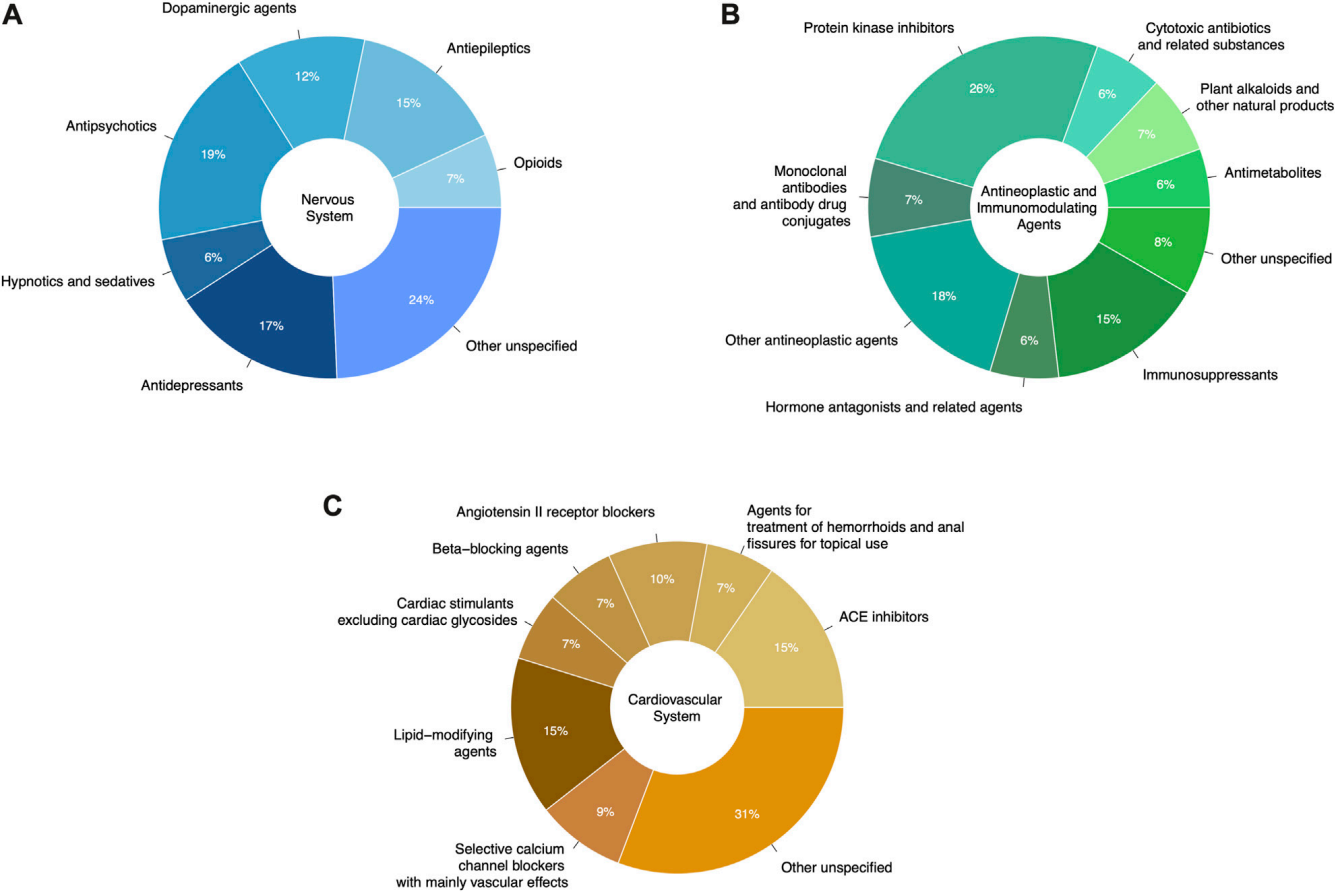
Further breakdown of unique prescribable drug candidates suggested for AD repurposing. **(A)** Proposed drugs acting on the nervous system (ATC code N). **(B)** Antineoplastic and immunomodulating repurposing candidates (ATC code L). **(C)** Repurposing candidates acting on the cardiovascular system (ATC code C). Drugs in classes accounting for less than 5% of the total drug count have been classified as “Other unspecified”.

Within distinct anatomical and pharmacological categories, there was still significant variability among the therapeutic subgroups of the suggested repurposing candidates, although some drug classes were more well-represented than others. For instance, antipsychotic medications (19%), antidepressants (17%), and antiepileptic agents (15%) represented around half of the suggested drug candidates acting on the nervous system (Figure 5A). Protein kinase inhibitors constituted 26% of the antineoplastic and immunomodulating drugs suggested as AD repurposing candidates, followed by other antineoplastic agents (18%) and immunosuppressants (15%) (Figure 5B). ACE inhibitors (15%), lipid-modifying agents (15%), and angiotensin II receptor blockers (10%) were among the most frequently suggested drugs with actions on the cardiovascular system (Figure 5C).

#### 3.1.3 Temporal changes in suggested drugs

**FIGURE 6:**
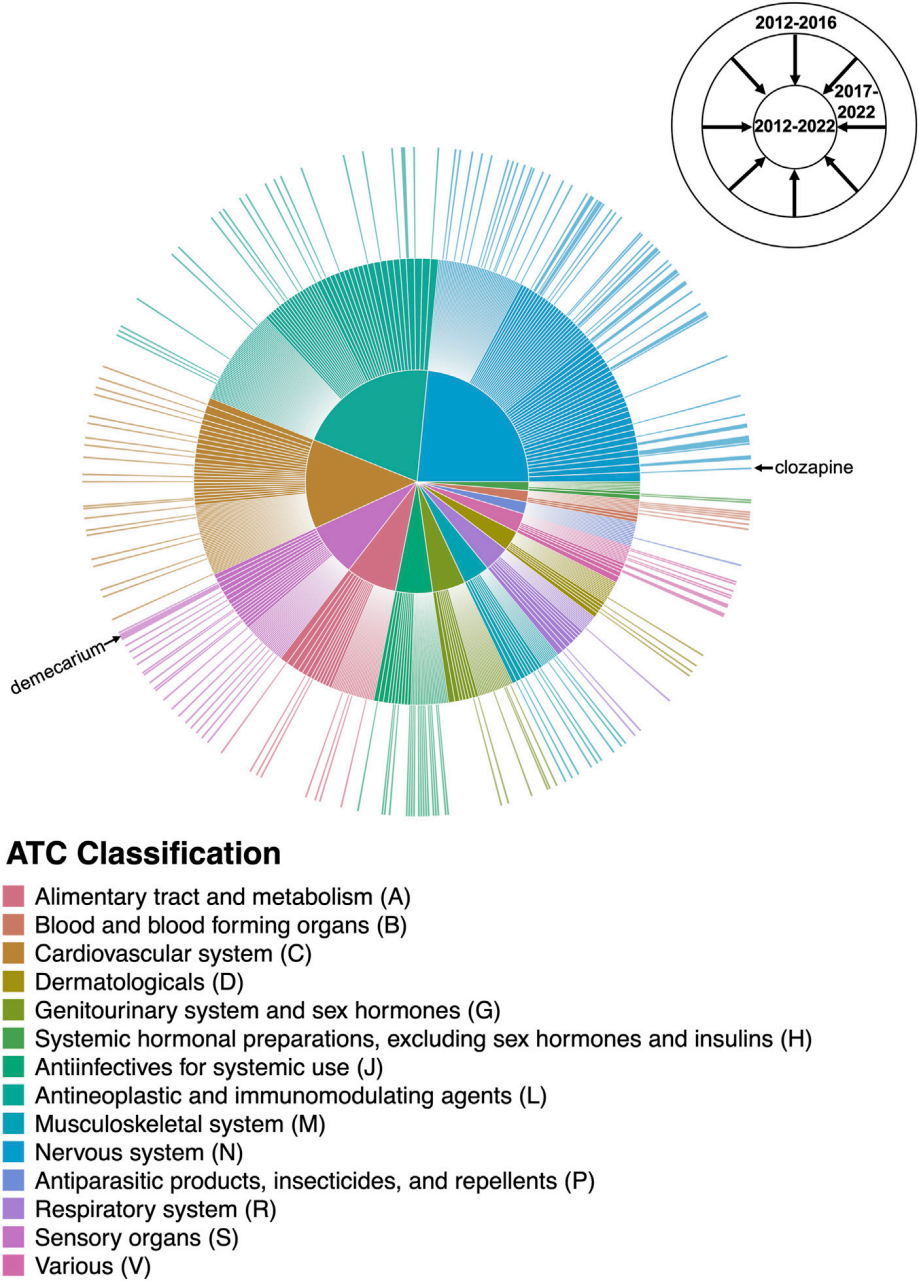
Changes in the AD drug repurposing landscape over time. The outermost ring of the sunburst plot represents drugs suggested by studies published between 2012 and 2016, with the size of each drug slice weighted by the number of unique supporting studies published during that time period. The innermost ring cumulatively adds drugs suggested by studies published in the last 5 years (between 2017 and 2022), providing a comprehensive breakdown of the AD repurposing candidates suggested between 2012–2022 and their literature support. Drug candidates are colored by their Level 1 ATC classification as shown in the center pie chart; for the purposes of this figure, drugs with more than one possible ATC code were mapped to a single code chosen at random. The drug slices corresponding to clozapine and demecarium are labeled. A high-resolution version of Figure 6 with all drug slices labeled is available as Supplementary Figure S1.

The expansion in the number and variety of AD repurposing candidates suggested between 2012 and 2022 is illustrated in Figure 6. Between 2012 and 2016, 184 unique prescribable drugs were proposed for repurposing in AD; between 2017 and 2022, 482 drugs were suggested. However, interestingly, only 93 drug candidates overlapped between the two time periods. Clozapine, the most frequently suggested AD repurposing candidate, is an example of one of such drugs with temporal continuity. Clozapine was initially proposed by a study published in 2016 (Issa et al., 2016), with subsequent support in five studies published between 2017 and 2022 (Choi et al., 2017; Chen et al., 2021; Chrétien et al., 2021; Gerring et al., 2021; Soleimani Zakeri et al., 2021). On the other hand, demecarium, a cholinesterase inhibitor used for topical treatment of glaucoma, is a representative example of a drug without temporal continuity—demecarium was first suggested as an AD repurposing candidate in 2014 (Croset et al., 2014), with subsequent support in two studies (Jamal et al., 2015; Zhang et al., 2016); however, after 2016, the drug was not mentioned again.

### 3.2 Validation strategies

61% (76/124) of the studies used additional data to validate the potential efficacy of their proposed AD repurposing candidates. Most of the experimental repurposing studies (45/48, or 94%) performed a validation, whereas only 44% (31/71) of the computational repurposing studies reported a validation.

**FIGURE 7:**
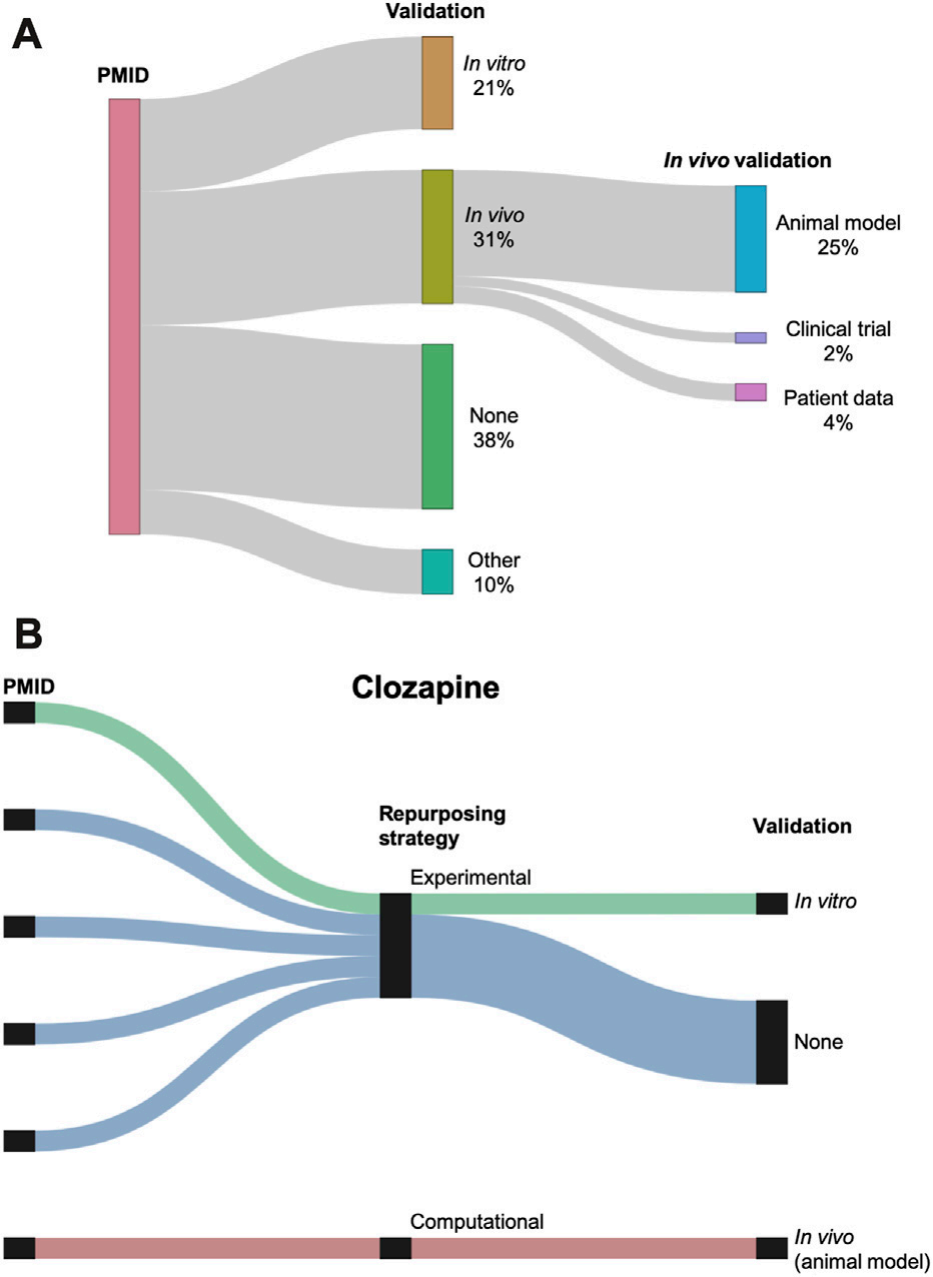
**(A)** Sankey diagram showing the breakdown of the validations performed across the studies. Validation is classified as *in vitro*, *in vivo*, other, or none. *In vivo* validation is further subdivided into animal models, clinical trials, and patient data. Three studies used two different types of validation (counted separately in the figure). **(B)** Breakdown of repurposing strategy and validation for the six studies suggesting clozapine as a repurposing candidate for AD. Repurposing strategy is classified as experimental or computational. Validation is classified as *in vitro*, *in vivo*, or none.

The 76 studies with validation employed a wide range of validation methods, categorized as *in vitro*, *in vivo*, and other (Figure 7A). *In vivo* validation in the form of animal models was the most popular validation method, used by 31 (25%) of the reviewed studies. Figure 7B shows the validation distribution for the six studies that suggested clozapine as a potential AD repurposing candidate. Only two of these studies performed a validation, one using *in vitro* data and one with *in vivo* data.

#### 3.2.1 Alzheimer’s-specific validation

**FIGURE 8:**
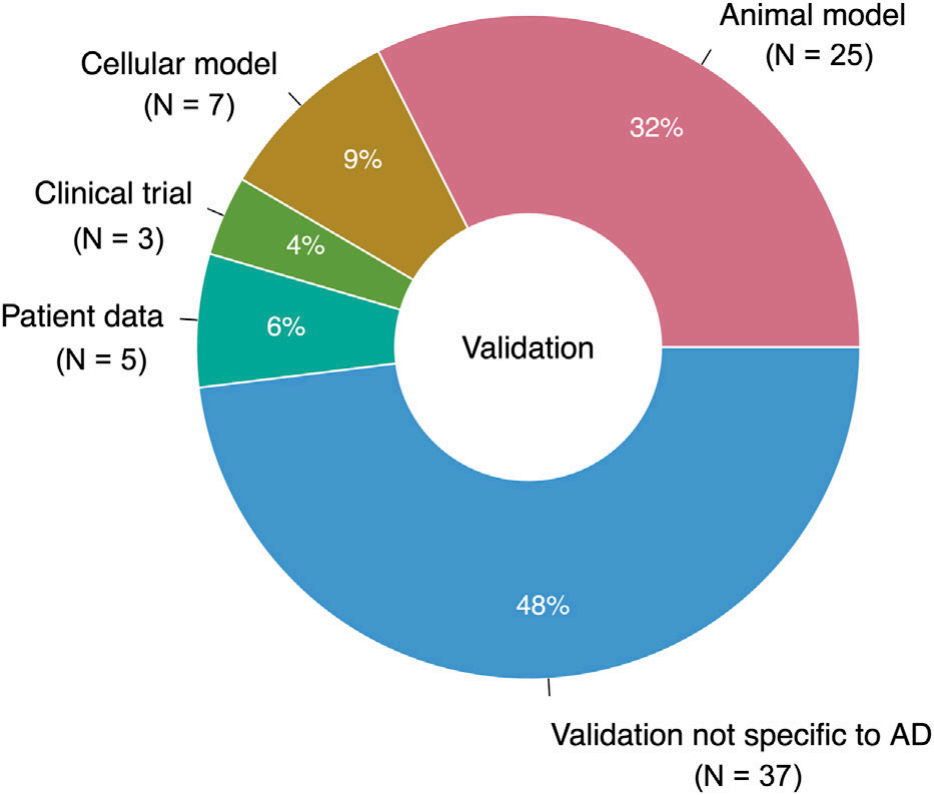
Breakdown of validation data types (AD-specific and nonspecific). 76 studies performed a validation; one study used two different types of AD-specific validation (N = 77 for entire pie chart).

Only 39 studies (31% of the total studies, or 51% of the studies with validation) performed validation using a system intended to reflect the characteristics of AD (Figure 8). Out of these studies with “AD-specific” validation, 25 used animal models of AD, neurodegeneration, neurotoxicity, or cognitive decline; seven used cell-based models; five used real-world data; and three were clinical trials. Together, the studies with AD-specific validation suggested 31 unique prescribable drugs. Only four drugs had two supporting studies with AD-specific validation: the antibiotic doxycycline, the nonsteroidal anti-inflammatory drug etodolac, the immunomodulatory drug fingolimod, and the antiemetic granisetron.

#### 3.2.2 Real-world data

Notably, only five studies (4%) utilized real-world data to validate their drug repurposing signals, and all were reported in the last 2 years (two published in 2021, three in 2022). Four of these studies performed pharmacoepidemiologic investigations, three leveraging the MarketScan Medicare Supplementary Material (Fang et al., 2021; Xu et al., 2021; Fang et al., 2022) and the fourth utilizing Medicare claims data (Desai et al., 2022). The fifth study used one EHR database to identify medications enriched among AD cases and controls, with validation in an independent EHR (Tang et al., 2022). Still another study used patient data from the Alzheimer’s Disease Neuroimaging Initiative ADNIMERGE dataset to identify potential drug repurposing candidates via association rule mining; however, the authors did not validate their findings in another clinical dataset (Prakash et al., 2021).

The AD repurposing candidates suggested and validated by the five studies using real-world data for validation included fluticasone and mometasone (inhaled corticosteroids) (Xu et al., 2021), dexamethasone (a systemic corticosteroid) (Tang et al., 2022), pioglitazone (an antidiabetic drug) (Fang et al., 2022), febuxostat (a uric acid-lowering drug used in treatment of gout) (Fang et al., 2022), atenolol (a beta blocker) (Fang et al., 2022), and sildenafil (a phosphodiesterase inhibitor used for treatment of erectile dysfunction) (Fang et al., 2021). One of the studies failed to validate their initial drug repurposing signal for disease-modifying antirheumatic drugs (Desai et al., 2022).

### 3.3 Role of journal impact

Finally, we explored the relationship between journal impact and signal quality to determine whether drug repurposing studies published in journals with higher impact factors were more likely to perform validation. Considering all journals with an impact factor available, we computed the mean impact factor for studies with validation compared to studies without validation. We found that studies without validation (N = 48) were published in journals with an average impact factor of 6.33 ± 3.61, while studies with validation (N = 75) were published in journals with an average impact factor of 6.17 ± 3.61. Using the categorized validation, we found that the studies with *in vivo* validation had an impact factor of 7.24 ± 4.27, whereas studies with *in vitro* validation had an impact factor of 4.94 ± 2.04. Studies with AD-specific validation were published in journals with only a slightly higher impact factor than studies without AD-specific validation (average impact factor 6.99 ± 3.94 compared to 5.89 ± 3.40).

## 4 Discussion

Drug repurposing for AD is an area of research that has experienced much growth throughout the last decade, with over 100 papers published since 2012. However, there remains minimal consensus among the hundreds of drug repurposing candidates that have been suggested for AD. Although drugs acting on the nervous system, antineoplastic and immunomodulating drugs, and drugs acting on the cardiovascular system represent general areas of highly suggested candidates, ultimately a wide range of medications with a variety of mechanisms of action and distinct therapeutic indications has accumulated, with considerable variability in the supporting evidence provided and no clear paths for prioritizing the suggested drugs.

### 4.1 Prioritizing the suggested drugs

Determining high-priority candidates remains a challenging task in drug repurposing for all diseases and was clearly demonstrated in this review. In terms of ATC classification, drugs acting on the nervous system (ATC code N, 17% of the suggested candidates) may be a particularly favorable class of drugs for AD repurposing—AD is a disease of the brain, and effective treatments for AD will need to cross the blood-brain-barrier. On the other hand, antineoplastic and immunomodulatory drugs may be less favorable for treatment of AD (ATC code L, 16% of the suggested candidates), given that many anticancer therapies and immunosuppressive agents carry significant adverse effects that may cause more harm than benefit in a long prescription period. However, these are still very broad categories, as demonstrated in Figure 5–drugs acting on the nervous system include antipsychotics, antidepressants, antiepileptics, and opioids; antineoplastic and immunomodulatory drugs encompass protein kinase inhibitors, monoclonal antibodies, hormone antagonists, and immunosuppressants, among others.

While the wide array of repurposing candidates for AD offers multiple potential therapeutic options to explore, it also presents challenges for identifying the most promising drugs among the many possibilities. Over half of the drugs (65%) had only a single study suggesting their utility in AD. Among all 124 reviewed studies, clozapine was most frequently proposed as a repurposing candidate for AD (N = 6); other frequently suggested drugs were aripiprazole, risperidone, sunitinib, vandetanib, vorinostat, pioglitazone, tamoxifen, verapamil, and adenosine (N = 5). Even among this high frequency subset, there is substantial variety—three are antipsychotics acting on the nervous system (clozapine, aripiprazole, risperidone); four are antineoplastic and immunomodulating agents (two protein kinase inhibitors: sunitinib and vandetanib, one hormone antagonist: tamoxifen, and one other antineoplastic agent: vorinostat); two act on the cardiovascular system (one calcium channel blocker: verapamil and one other cardiac preparation: adenosine); and one acts on the alimentary tract and metabolism (the blood glucose lowering drug pioglitazone). However, when using validation as an indicator of high-quality drug repurposing signals, the list changes—the most frequently suggested drugs with AD-specific validation were doxycycline (an antibiotic), etodolac (a non-steroidal anti-inflammatory drug), fingolimod (an immunomodulating medication used in the treatment of multiple sclerosis), and granisetron (used as an antiemetic to treat nausea and vomiting) [N = 2]. Further, when focusing solely on the drugs with real-world data validation, yet another list of drugs emerges: fluticasone, mometasone, dexamethasone, pioglitazone, febuxostat, atenolol, and sildenafil. The only point of overlap between these three lists is the antidiabetic drug pioglitazone.

#### 4.1.1 Antipsychotics

Among the suggested AD drug repurposing candidates acting on the nervous system, 19% were antipsychotics, including clozapine, aripiprazole, and risperidone. While the precise mechanisms of these drugs are largely unknown (Seeman, 2002), they are thought to act primarily on dopaminergic (D2) and serotonergic (5-HT2A) receptors, with additional effects on adrenergic, cholinergic, and histaminergic receptors. Many of the existing drugs for AD are directed at neurotransmitters, namely, acetylcholine (i.e., donepezil, rivastigmine, galantamine) and glutamate (i.e., memantine), thus it is possible that antipsychotic medications have a higher frequency of suggestion due to an overlap in neurotransmitter imbalances between schizophrenia and AD. Despite their high frequency of suggestion, however, there has been limited investigation of antipsychotic medications in clinical trials of AD. Neither clozapine nor aripiprazole have been studied in clinical trials of AD reported in ClinicalTrials.gov. While several trials have investigated risperidone use in AD, this has been in the context of alleviating behavioral and psychological symptoms (e.g., agitation, hallucinations, and delusions) observed in some individuals with AD, rather than as a potential treatment for AD (Brodaty et al., 2003; Mintzer et al., 2006). The limited clinical investigation of antipsychotics in AD may be related to their adverse effects, which include increased risk of cardiovascular and cerebrovascular events, metabolic syndrome, extrapyramidal symptoms, and for clozapine specifically, agranulocytosis, and may be more severe in older adults. (Stroup and Gray, 2018).

### 4.2 Variability in validation

We found that validation was inconsistently performed among the reviewed studies, with only 61% (76/124) of the studies conducting additional investigations to support their preliminary drug repurposing candidates. Animal models and *in vitro* studies were most commonly used for validation, which may explain the particularly high validation rate among the experimental studies (94%). Interestingly, no combination studies performed a further validation, although this may have been an artifact resulting from how we defined this repurposing strategy (computational and experimental studies performed in tandem to suggest two different types of drugs, rather than performed sequentially to filter down a drug list).

#### 4.2.1 Alzheimer’s-specific validation

Among the studies with validation, only roughly half (51%) were determined to have AD-specific validation. This is likely due to the broad definition of AD applied in this study—again, we included studies that developed general drug repurposing tools and applied them to AD, as well as studies suggesting repurposing candidates for diseases including but not limited to AD. We observed that many of the studies with nonspecific validation relied on mechanistic hypotheses about AD, particularly in investigating molecular targets of AD. These molecular investigations included well-known targets such as amyloid β (Aβ), acetylcholinesterase (AChE), and β-secretase (BACE-1), but also several lesser known targets such as EPHA4 (Gu et al., 2018) and MARK4 (Hruba et al., 2022). There was a very slight difference in journal impact factors for studies with and without AD specific validation (6.99 vs 5.89), suggesting that the journal of publication is of little use in distinguishing high-quality drug repurposing signals. However, we also acknowledge that our analysis of AD-specific validation was limited by the lack of existing comprehensive experimental models for AD (Drummond and Wisniewski, 2017).

#### 4.2.2 Limited use of real-world data

Real-world data, defined as patient health data from non-clinical trial sources, were surprisingly rarely used for validation of drug repurposing candidates (4%). Four of the studies used health insurance claims data (Fang et al., 2021; Xu et al., 2021; Desai et al., 2022; Fang et al., 2022), and one study leveraged data from two large-scale independent EHR databases (Tang et al., 2022). The use of real-world data to investigate drug repurposing signals has become an appealing approach (Xu et al., 2015; Park, 2021; Liu and Panagiotakos, 2022; Zong et al., 2022), particularly in light of the 21st Century Cures Act enacted in 2016, which set forth a framework for use of real-world evidence in drug repurposing (Center for Drug Evaluation and Research, 2021). Real-world data has certain advantages, notably larger sample sizes, more representative patient populations, increased speed of investigation, and lower costs compared to clinical trials. EHRs represent a particularly promising source of real-world data with rich longitudinal medication information tied to real-time clinical outcomes that can be leveraged to confirm (or disprove) the expected effects of repurposing candidates. The full potential of real-world data in drug repurposing for AD should be thoroughly explored in future studies, as drugs with promising signals may represent strong repurposing candidates warranting further investigation in clinical trials.

### 4.3 Negative and contradictory findings

The large number of AD repurposing studies presents possibilities for negative and contradictory results, which must be carefully considered. Only one of the reviewed studies reported evidence of increased AD risk associated with drug use, specifically for the PCSK9 inhibitors evolocumab and alirocumab (Williams et al., 2020). These results were not directly contradicted by any of the other reviewed studies, which did not suggest either evolocumab or alirocumab as potential AD repurposing candidates. Several studies reported evidence of drug candidates likely to have little to no effect on AD risk. These drugs included lipid-lowering agents (ezetimibe, mipomersen, and statins) (Williams et al., 2020), antihypertensives (Walker et al., 2020), dimethyl fumarate (approved for the treatment of multiple sclerosis) (Möhle et al., 2021), and drugs commonly used in treatment of rheumatoid arthritis (tofacitinib, tocilizumab, and TNF inhibitors) (Desai et al., 2022). Still, statins (atorvastatin, simvastatin, rosuvastatin, fluvastatin) and ezetimibe were suggested as viable AD repurposing candidates by other studies, suggesting that reported negative outcomes may not entirely discredit the candidacy of certain drugs (Cheung et al., 2014; Croset et al., 2014; Kondo et al., 2017; Bauzon et al., 2020; Wu et al., 2021).

### 4.4 Limitations

Our study has several limitations. Importantly, we focused our literature search on PubMed, which is largely regarded as the primary database for biomedical literature. As a result, we may have missed some AD drug repurposing candidates discussed in journals from other fields, such as computer science (e.g., IEEE Journals, which are indexed by Google). In addition, we found that very few studies reported explicitly negative outcomes (i.e., a drug candidate found to have paradoxical effects or even no effect), suggesting potential selective reporting bias, which may have impacted our results.

### 4.5 Challenges

Given the large number of AD drug repurposing studies and suggested repurposing candidates, consolidating the evidence to identify high-priority drugs for subsequent investigation in clinical trials has proven to be a major challenge. Validation represents one approach to prioritizing among the suggested drugs, as the drugs with high-quality validation data should intuitively be more promising repurposing candidates. However, as demonstrated in this study, validation is inconsistently performed—39% of the reviewed studies did not perform a validation, and the studies that did perform a validation used a variety of different approaches, roughly half of which had limited relevance to AD. Furthermore, the complexity of the mechanisms underlying the pathogenesis of AD and its clinical heterogeneity have made it incredibly difficult to develop robust models of AD for use in validation, potentially limiting the generalizability of the drug repurposing studies that sought to perform AD-specific validation. Finally, the clinical viability of the suggested drugs requires deeper exploration, as many studies neglected to consider possible safety concerns associated with drug use when providing repurposing candidates (e.g., immunosuppressants and antineoplastic agents).

### 4.6 Future directions

Despite its challenges, drug repurposing for AD still holds much promise, particularly in expanding the scope of possible AD therapies beyond drugs targeting amyloid β. However, future drug repurposing studies will need to make a concerted effort to narrow down the list of candidates, which will require thorough validation. Real-world data, particularly from EHRs, represents an especially valuable tool for investigating long-term drug effects in real-time patient health outcomes but was rarely used for validation in the reviewed studies. Going forward, leveraging this big data from diverse datasets (e.g., the National Institutes of Health *All of Us* Research Program, UK Biobank, and local EHRs) will be critical to identify clinically meaningful drug repurposing candidates for AD. The incorporation of EHR data into drug repurposing pipelines may also transform the process of identifying drug repurposing candidates, transitioning from primarily hypothesis-driven studies to non-hypothesis-driven approaches relying on pattern identification in large EHRs.

## 5 Conclusion

Given limited successes in new drug development for AD, there has been growing research interest in finding existing drugs that can be repurposed for AD treatment. Between 2012 and 2022, 124 studies cumulatively suggested 573 drugs as potential AD repurposing candidates. However, identifying the most promising candidates remains a challenging task, as the suggested drugs vary widely in terms of their therapeutic indications and studies do not consistently validate preliminary drug repurposing signals. Importantly, real-world data has seldom been used to validate AD drug repurposing candidates, despite the enormous potential of EHRs and other large-scale repositories of clinical data to confirm the expected treatment effects of suggested drug repurposing candidates. Future AD drug repurposing studies should aim to establish best practices for validation, including investigating opportunities for leveraging EHRs and other sources of real-world data to prioritize among suggested drug candidates.
